# Molecular Mechanisms and Potential Therapeutic Targets of Ischemia–Reperfusion Injury in Kidney Transplantation

**DOI:** 10.3390/cimb47040282

**Published:** 2025-04-17

**Authors:** Aaron J. Huang, Gaurav K. Sharma, Rohan Parikh, Zhaosheng Jin, Frank S. Darras, Sergio D. Bergese

**Affiliations:** 1 Department of Urology and Transplant Surgery, Stony Brook University Hospital, Stony Brook, NY 11794, USA; aaron.huang@stonybrookmedicine.edu (A.J.H.); frank.darras@stonybrookmedicine.edu (F.S.D.); 2 Renaissance School of Medicine, Stony Brook University, Stony Brook, NY 11794, USA; gaurav.sharma@stonybrookmedicine.edu (G.K.S.); rohan.parikh@stonybrookmedicine.edu (R.P.); 3 Department of Anesthesiology, Stony Brook University Hospital, Stony Brook, NY 11794, USA; zhaosheng.jin@stonybrookmedicine.edu

**Keywords:** end-stage renal disease, kidney transplant, ischemia, reperfusion injury, donor, inflammation, machine perfusion, graft

## Abstract

End-stage renal disease (ESRD) is a serious and lethal disease that carries with it a high morbidity and mortality rate if left untreated. Treating ESRD is conducted via renal replacement therapy and/or kidney transplantation, with the latter being the preferred option given the better outcomes and quality of life for the patients. However, as ESRD rises in prevalence, kidney transplantation rates remain largely unchanged. In every kidney transplantation, ischemia–reperfusion injury (IRI) is inevitable and the effect this has on the kidney depends based on donor type. IRI works through a variety of molecular mechanisms, primarily mitochondrial oxidative stress and programmed cell death mechanisms. Given the urgency to ensure the best outcomes for these limited kidney transplants, there has been a continued effort to find various potential therapeutic mechanisms to counteract IRI preoperatively, intraoperatively, and postoperatively. These include hypothermic machine perfusion, ischemic conditioning, nanoparticle removal of free radicals, peptide-based therapies, microRNA, and more. There is an ongoing effort to find the best way to mitigate IRI in kidney transplantation and this is being achieved through a better understanding of the molecular mechanisms of IRI.

## 1. Introduction

End-stage renal disease (ESRD) or kidney failure is when the kidneys lose their ability to function effectively over time, with the only available treatments being dialysis or kidney transplantation [1]. A global prevalence study of ESRD in 2010 found up to 10 million people in the world requiring treatment [2], with the rates of kidney failure in the US being almost double or three times higher than those reported in other developed countries [3,4]. The lifetime risk of developing ESRD was cited to be as high as 1 in 50 by age 40 [5]. The prevalence of ESRD rises with age with older patients (>65 years old), representing the fastest growing population segment starting dialysis [6]. Current trends continue to show the rising prevalence of ESRD [2] due to an aging population with more comorbidities also being on the rise. This emphasizes the importance of addressing ESRD and having a better understanding behind its severity and its treatment modalities.

Given the vital physiologic roles of the kidney in balancing electrolytes, blood pressure, blood count, and fluid/waste management, ESRD is associated with significant morbidity and mortality [1,5,7]. In a study from Mexico, researchers found an almost 70% mortality rate for patients with ESRD that did not start dialysis, with a nearly 50% decrease in mortality rate if patients started dialysis in 90 days [8]. A more global study by Thurlow found the unadjusted 5-year survival rates of ESRD patients, even while on treatment, to be 40–60%, with the United States around 41% [2]. These studies are only a couple from the expansive set of literature available, but highlight how important receiving treatment is for patients with ESRD given its high mortality rate.

Treatment options for ESRD include renal replacement therapy (peritoneal or hemodialysis) and kidney transplantation. The treatment chosen for the patient is often influenced by access to treatment, treatment outcomes, and effect on quality of life. Despite kidney transplantation being widely accepted as having better long-term outcomes in terms of both patient outcome [4,5,7,9,10,11] and quality of life [7,11], dialysis ends up typically being the main treatment option due to the limited availability of kidneys to transplant [2]. The United States has the highest transplantation rate in epidemiological studies, showing a rate of 75 per million of the population [4]. Even so, the donation rate of kidneys has continued to consistently lag behind demand [5]. This fact, coupled with the fact that ESRD continues to rise, shows the importance of making sure each transplant functions correctly in as ideal a setting as can be provided medically. There continues to be improvement in graft survival, with 1- and 5-year graft survival rates up to 97.8% and 86.5%, though this differs based on donor type [12].

Kidney transplantation is the practice of implanting either a deceased or live donor’s kidney into the recipient, with wait times to receive a transplant being 3 to 5 years on average worldwide. Transplanted kidneys come from a few types of donors: living, circulatory death, and brain death donors. With each donor type, a big difference is the ischemia time, and, with that, the resultant graft survival and function of the graft, as delineated in Table 1 [12,13,14,15]. While donors from brain death individuals have, at times, comparable ischemia times, a UK team specifically allocated those kidneys to farther areas compared to the DCD donors given that DBD donor kidneys were less susceptible to ischemia–reperfusion injury (IRI) [13,14]. Though there are large ranges of ischemia times and graft survival statistics cited from these studies due to the operating protocols of different countries along with their respective graft preservation strategies and year of cited publication, Table 1 illustrates an important point. It highlights the consistent findings that a greater ischemia time can affect delayed graft function (DGF) and overall graft survival, especially on DCD donor kidneys [14].

## 2. Ischemia–Reperfusion Injury and Its Molecular Mechanisms

Ischemia–reperfusion injury is a type of acute kidney injury which occurs after the tissue is reperfused after a period of ischemia time, inducing biochemical processes that can cause further injury to the graft [16]. Given that graft explantation involves the disruption of its blood supply, some degree of ischemia is an unavoidable part of the process. The ischemia differs based on the situation or where the kidney is coming from, as donor type is strongly associated with the severity of the injury [17]. This phenomenon of IRI in kidney transplantation has been widely known and continues to be a hotbed of innovation given, as mentioned, the sheer prevalence of end-stage renal disease and the stagnant number of organ donors. To understand how this is being ameliorated, it is paramount to understand the various molecular mechanisms studied and proposed to influence IRI.

### 2.1. Mitochondrial Oxidative Stress, Immune, and Inflammatory Responses in IRI

The kidney has an abundance of ATP-dependent physiological functions, which requires large amounts of oxygen for aerobic energy production. Along with the heart, the kidney has the highest resting metabolic rate and the second highest oxygen usage and mitochondrial content. In the setting of ischemia during kidney transplantation, there is a switch from aerobic to anaerobic metabolism with reduced ATP production and increased lactate production. This underlies the initial mechanism of renal ischemic injury: dysfunction of Na^+^/K^+^ ATPases, Na^+^/H^+^, and Ca^2+^-ATPase pumps; the subsequent accumulation of sodium, hydrogen, and calcium in the cytoplasm; and ultimately, cellular swelling as water is transported into the cell. Reperfusion rapidly returns oxygen and pH levels to their normal levels, triggering a rapid influx of calcium into the cell and stimulating the apoptosis pathway via the activation of cysteine proteases and the release of pro-apoptotic/necrotic factors from the mitochondria [18,19].

A key driving factor of cell injury in IRI is mitochondria metabolism, which produces >90% of intracellular reactive oxygen species (ROS). ROS are produced by the deregulation of enzymes like NADPH oxidase (NOX), nitric oxide synthase (NOS), the electron transport chain, and xanthine oxidoreductase (XOR). NOX complexes can be activated by factors released during IRI such as hypoxia inhibitory factor-1α (HIF-1α), phospholipase A2, arachidonic acid, complement system, and cytokines (TNF-α, IL-1β) from immune cells. NOS, in hypoxic conditions, can be converted from producing NO into an O_2_-generating enzyme. Finally, mitochondria also directly produce ROS by the univalent reduction of O_2_, with the major source of mitochondrial superoxide arising at respiratory complex I [18,19,20,21].

One mechanism by which oxidative stress can cause mitochondrial injury is by triggering excess mitochondrial fission [20] and mitophagy. This mechanism is mediated by dynamin-related protein 1 (Drp1), which upregulates mitochondrial fission and dysfunction and leads to mitophagy. One study showed that Drp1 was activated by S616 phosphorylation and S637 dephosphorylation and translocated to the mitochondria after renal IRI. When inactivated by direct Drp1 inhibition with mitochondrial division inhibitor-1 (mdivi-1) or by blocking the mitochondrial translocation of Drp1 by mitochondrial fission 1 (Fis1), the IRI-induced loss of mitochondria was at least partially rescued [22]. Furthermore, a study by Lang et al. showed that this mechanism may have been mediated by miR-199a-5p, which was upregulated in transplanted mouse kidneys and human kidney DGF samples. This microRNA (miRNA) exerts its effects by downregulating A-kinase anchoring protein 1 (AKAP1) and S637 dephosphorylation of Drp1 [23]. Deletion of Drp1, specifically in the proximal tubule of mouse kidneys, has also been shown to prevent IRI-induced kidney injury, inflammation, and apoptosis, as well as progression to fibrosis and dysfunction. This highlights its role in inducing fission and mitophagy, key drivers in mitochondrial injury during IRI [24].

Mitochondrial DNA (mtDNA) damage is another hallmark characteristic, alongside the production of mitochondrial ROS (mtROS), as significant contributors to mitochondrial dysfunction and inflammation in renal IRI. One important mechanism linking these features is mtROS-induced TFAM (mitochondrial transcription factor A) depletion in tubular epithelial cells (TECs), specifically by a reduction in TFAM transcription and degradation by the upregulation of Lon protease activity. Loss of TFAM causes mitochondrial dysfunction in TECs by increasing levels of mtROS and reducing respiration and ATP production. Furthermore, TFAM and mtDNA nucleoids stabilize each other by binding; thus, a loss in TFAM causes a subsequent loss of stability of mtDNA nucleoids and a loss of mtDNA synthesis. This process results in impaired mitochondrial energy production, and therefore, renal dysfunction. The impact of mtROS on TFAM also induces the inflammatory response caused by the release of endogenous damage-associated molecular patterns. The loss of TFAM can induce the aberrant packaging of nucleoids, causing mtDNA to leak into the cytosol and produce cytokines. The release of cytokines can cause macrophage infiltration and other inflammatory processes [25].

### 2.2. Programmed Cell Death

Apoptosis typically refers to caspase-dependent processes; however, recent processes have shown caspase-independent processes that work through signaling pathways. Newly identified programmed cell death pathways, including pyroptosis, necroptosis, and ferroptosis, are important mechanisms mediating renal IRI, and have been well described in Li et al.’s 2024 review [26]. In particular, ferroptosis causes a “wave of death” phenomenon in which synchronized cell death occurs in adjacent cells. One paper partially elucidated the mechanism underlying it by revealing the role of the downstream products of phospholipid oxidation, platelet-activating factor (PAF) and PAF-like phospholipids (PAF-LPLs), in initiating and mediating ferroptosis by increasing lipid bilayer permeability and causing membrane instability. In addition, PAF and PAF-LPLs are involved in the propagation of cell death to damage whole renal tubules through rapid generation at high concentrations and expulsion from damaged cells, both in secreted micro-vesicle particles and in free form [27]. Another mechanism underlying this phenomenon involves renal IRI cell-secreted small extracellular vesicles (IRI-sEVs), which deliver lncRNA-WAC-AS1 and in turn induce GFPT1 expression. This leads to an increase in BACH2 O-GlcNAcylation in neighboring cells, transcriptionally suppressing proteins that protect against ferroptosis such as SCL7A11 and GPX4 [28]. This propagation through IRI-sEVs also may play a role in the transition of AKI-to-CKD [29].

Necroptosis is primarily characterized by receptor-interacting protein kinases 1 and 3 (Rip1/Rip3) and mixed lineage kinase domain-like pseudokinase (MLKL), which form the necroptosome [30,31]. In vitro and in vivo mouse kidney models have shown that Rip1/Rip3 are present in kidney tissue and that Rip1 mediates TNF-α-mediated necroptosis. Furthermore, in vivo mouse models of renal IRI treated with the Rip-1 specific inhibitor necrostatin-1 (Nec-1) experienced a protective effect against IRI, while mice treated with apoptosis-blocking pan-caspase inhibitor zVAD did not [31].

These processes all represent different deadly outcomes of a connected system; however, they offer avenues for research on potential therapeutic targets as well.

### 2.3. Inflammatory and Immune Responses

The inflammatory and immune responses to renal IRI have been well described in several reviews covering the innate immune response [32], adaptive immunity through B and T cells [33,34], and especially the complement system [35]. Some specific, unique mechanisms mediating the early immune response, especially by T cells, have been investigated and may provide novel therapeutic options.

T lymphocytes play a role in the early phase of IRI, and may be specifically mediated by the Fas/Fas ligand (FasL) pathway. In a mouse model of bilateral renal IRI, FasL knockout was protective after 24 h and had a lower production of TNF-α by the T cells. The FasL specific to leukocytes, rather than non-hematopoietic cells, was majorly responsible for its harmful effect in renal IRI. Importantly, FasL knockdown also reduced cytokine production after IRI, a key step in recruiting further inflammation-inducing cells [36]. Another mechanism that may play a role in inducing CD4+ and CD8+ T cell proliferation involves short-chain fatty acids (SCFAs) produced by the gut microbiome as end products of fermentation. SCFAs were shown in a mouse model to improve kidney function after renal IRI, with acetate decreasing ROS production and reducing cytokine production; this was associated with lower levels of neutrophils and macrophages. In addition, treating antigen-presenting cells with LPS and SCFAs led to the lower proliferation of CD4+ and CD8 T cells [37].

## 3. Potential Therapeutic Targets and Surgical Considerations

**Figure 1:**
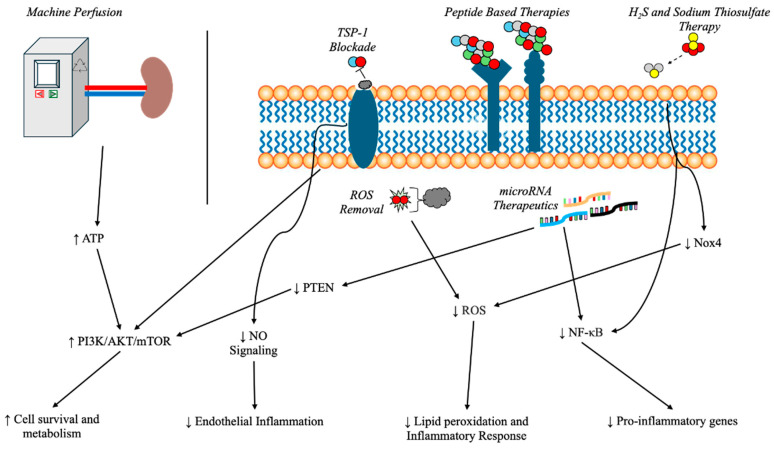
Potential therapeutic strategies to ameliorate ischemia–reperfusion injury in kidney transplantation.

Renal graft injury, particularly IRI, remains a principal challenge in kidney transplantation due to its complex molecular and cellular underpinnings. Although advances in immunosuppression and surgical techniques have improved transplantation outcomes, the development of targeted therapies to prevent or mitigate IRI remains elusive. Recognizing the critical role of kidney transplantation for patients with end-stage renal disease, research continues to unveil novel molecular mechanisms and therapeutic strategies for graft preservation. This section discusses the emerging and current targets and interventions aimed at improving renal graft survival and function (Figure 1).

### 3.1. Cold Storage vs. Machine Perfusion

Preservation strategies are integral to successful renal transplantation, with static cold storage (SCS) and machine perfusion (MP) being the two principal approaches. SCS involves hypothermic preservation to slow cellular metabolism, thereby reducing ischemic damage. By contrast, MP circulates a preservation solution through the organ, promoting oxygenation and the removal of metabolic byproducts. There are many widely used perfusate solutions that vary in electrolyte content, osmolarity, impermeants, antioxidants, and other additives. These all function to optimize the kidney’s nutrients and oxygen reserves while being under MP—to mimic the intracellular environment and prevent cellular edema and oxidative stress [38]. Studies have consistently shown that MP significantly lowers DGF and enhances long-term graft survival relative to traditional SCS [39,40,41]. In a multicenter randomized controlled trial, machine-perfused kidneys demonstrated superior early post-transplant function and a reduced incidence of primary non-function [39]. Meta-analyses of sixteen studies also showed the superiority of MP over SCS for deceased donor kidney transplantation, as it significantly reduces DGF, leading to better short-term and potential long-term graft outcomes [42].

One potential mechanism by which MP protects allografts is by the protection of endothelial cell function. In a porcine model, MP specifically caused the phosphorylation and activation of endothelial NO synthase in an AMPKα-dependent manner. Studies of the renal arteries ex situ after reperfusion and the transplanted kidneys in vivo further showed that MP protects allografts and significantly improves early cortical microcirculation in an NO-dependent mechanism [43,44]. Given the clear benefit of MP over SCS, there has been research conducted in regard to the temperature with MP to further enhance long-term graft survival.

Normothermic MP maintains the organ at physiological temperatures while providing oxygen and nutrients. One study showed that normothermic MP an hour prior to transplantation is a feasible and safe preservation method for donation after circulatory death (DCD) kidney transplantation, though this was a non-randomized pilot study [45]. The same group ten years later performed a randomized controlled trial, which found that a 1 h normothermic MP just before transplantation did not significantly reduce DGF compared to SCS. However, they also noted difficulty in standardizing this 1 h perfusion window, as some kidneys had to be re-placed into SCS for a couple more hours, warranting further investigation into optimal perfusion strategies as that may have affected results [46].

Hypothermic MP (HMP) also has the potential to reduce the thrombo-inflammatory response immediately following reperfusion in IRI. In a cohort study from Strandberg et al., deceased donor renal transplants preserved with SCS showed the sustained coactivation of the complement, coagulation, and kallikrein–kinin systems, while those preserved with HMP showed an attenuation of this response. Clinically, this reduction in the thrombo-inflammatory response was associated with a preservation of the eGFR 24 months following transplantation [44]. Another mechanism by which HMP exerts its protective effect in renal IRI is by modulating A20. A20, a target gene of NF-κB, participates in a negative feedback mechanism to regulate NF-κB expression. In a rabbit model, kidneys that underwent HMP had significantly increased A20 expression relative to SCS due to reduced levels of MALT1, which cleaves A20, although the mechanism by which HMP reduces MALT1 has not been elucidated. A20 was found to protect kidneys by suppressing inflammation, apoptosis, and necroptosis. The anti-inflammatory effect arose via A20 expression in TECs reducing NF-κB and TNF-α expression. Anti-apoptotic effects were specifically mediated by suppressing the ASK1-JNK signaling cascade. Concomitant with the increased A20 expression, HMP kidneys exhibited a reduced expression of ASK1, p-JNK, and cleaved caspase-3, and a lower rate of apoptosis. Finally, A20 expression during IRI also specifically restricted RIPK3-dependent necroptosis [47].

### 3.2. Ischemic Conditioning

After preoperative management to reduce IRI, there was a promising intraoperative technique that could have potentially reduced IRI in the form of ischemic conditioning. Ischemic conditioning is the practice of applying one or many brief periods of ischemia and reperfusion to induce tolerance to subsequent episodes of ischemia and reperfusion [48]. This was first introduced in dog studies to ameliorate cardiac ischemic episodes and has been translated to renal transplantation with mixed results. It has been suggested that the mechanism of ischemic conditioning is a systemic response, and that even a remote ischemic stimulus may induce a general protective response to further episodes of IRI [49,50].

Remote ischemic preconditioning was first performed in a rat model where repetitive sequences of ischemia and reperfusion were performed via a cuff on their hind leg [49]. This was repeated in a porcine model showing only a slight protective effect at 24 h with no lasting changes at 3 to 7 days [51]. These mixed results persisted when translated to human studies. A study by Wu et al. found that 3 × 5 min cycles of ischemia–reperfusion via clamping of the external iliac artery prior to transplantation found an improvement in Cr and eGFR compared to the control group up to postoperative day 14, though noted this to not be significantly different by 30 days [50]. This was subsequently followed up by another study, but on living donor recipients, which found there to be no protective effects from remote preconditioning [52]. A rat model of postconditioning found that ischemic conditioning at the onset of reperfusion after an ischemic stimulus attenuated IRI and decreased apoptosis [53]. Given such promise in animal models, ischemic conditioning was then brought to clinical trials with mixed results. Zhang et al.’s group was the first to be published, which found no reduction in the incidence of DGF, but remote ischemic conditioning did lead to increased 3-month eGFR, a proxy of graft function [54]. However, two subsequent meta-analyses, published months later, were not able to confirm these findings, and in fact showed no difference in DGF and eGFR [55,56]. There appears to be some promise in remote ischemic conditioning, both pre- and post-, to potentially alleviate DGF, but may not affect overall graft survival, though this continues to be an intriguing aspect of intraoperative management to mitigate IRI.

### 3.3. MicroRNA as Therapeutic Targets

MicroRNAs (miRNAs) are small, non-coding RNAs that regulate gene expression post-transcriptionally, influencing processes such as inflammation, fibrosis, and immune activation. In renal transplantation, specific miRNAs have been implicated in the pathogenesis of IRI [57,58]. miR-210 and miR-155 are upregulated during IRI, with miR-155 activating the NF-κB pathway and suppressing SOCS1, leading to an exaggerated inflammatory response and increased liver damage [58]. In contrast, miR-122, miR-30b, miR-20a, miR-125b, miR-494, miR-148a, and miR-93 are downregulated, with protective effects such as inhibiting NF-κB, activating the PI3K/Akt pathway, and reducing TRAF6- and TLR4-mediated pro-inflammatory signaling, suggesting potential therapeutic targets to mitigate IRI [58]. Another review highlighted miR-21’s protective role in IRI via the PTEN/Akt/mTOR/HIF pathway, alongside miR-155, miR-210, miR-126, miR-192, miR-423, and miR-93, which regulate inflammation, endothelial function, and AKI progression [57]. There was even a clinical trial, albeit on Alport Syndrome, of the anti-miR-21 drug lademirsen, which unfortunately failed to find any improvement in kidney function decline in a phase 2 study [59]. Recently, renal unilateral IRI mice models and H/R NRK52E cell assays were used and showed that miR-378 was found to reduce apoptosis, inflammation, and fibrosis by targeting caspase 3, with agomir-378 treatment improving tubular integrity and decreasing immune cell infiltration, suggesting its potential as a therapeutic target for renal allograft protection [60]. Ongoing advances in RNA therapeutics highlight the feasibility of leveraging miRNAs as novel therapeutic agents in transplantation.

### 3.4. Peptide-Based Therapies

Peptide-based therapeutics have gained attention for renal graft protection due to their high specificity, favorable pharmacokinetic profiles, and relatively low toxicity. These agents target critical pathways linked to IRI, such as inflammation and endothelial dysfunction. Synthetic peptides mimicking endogenous proteins (e.g., thrombomodulin and annexin A1) have been shown to reduce inflammation, mitigate tubular necrosis, and promote tissue repair [61]. In a murine model of kidney transplantation, a novel peptide-based treatment significantly decreased acute kidney injury and improved long-term allograft survival through the modulation of inflammatory cascades [61]. With continuous refinements in peptide design and delivery, these therapies hold some promise as adjuncts to current transplant regimens, though clinical trials have yet to be published on these therapies.

### 3.5. Thrombospondin-1 Blockade Using Nanoparticles

Thrombospondin-1 (TSP-1) is a glycoprotein known to inhibit nitric oxide signaling, contributing to endothelial dysfunction and immunological activation following transplantation. Elevated TSP-1 levels correlate with increased endothelial damage and a heightened risk of graft rejection. Another signaling pathway, TSP-1, has been found to be involved in Drp-1 and ROS generation, with a study on rat heart tissue finding that the inhibition of TSP-1 prevents Drp-1-mediated mitochondrial fission, thus reducing the cellular damage from IRI [62]. Recent nanoparticle-based delivery systems have enabled the targeted inhibition of TSP-1 within the transplanted kidney, preserving vascular integrity and reducing ischemic insults [63]. In murine models, TSP-1 blockade via nanoparticles significantly diminished immune cell infiltration and improved graft survival, underscoring the therapeutic potential of inhibiting TSP-1 in renal transplantation.

### 3.6. Reactive Oxygen Species (ROS) Removal via Nanoparticles

Reactive oxygen species (ROS)-responsive nanoparticles offer another approach to mitigating IRI by directly scavenging oxidative stress, reducing inflammation, and preserving cellular integrity. Among the most effective strategies, ceria nanoparticles (CeO₂ NPs), polyoxometalate nanoclusters, and framework nucleic acids (FNA) neutralize ROS, protecting against oxidative damage and apoptosis [18,64]. DNA origami nanostructures (DONs) and selenium-doped carbon quantum dots (SeCQDs) further enhance redox balance, while some nanosystems serve as carriers for anti-inflammatory agents like anti-IL-6 antibody and rapamycin to suppress secondary injury [64]. Additionally, targeting ferroptosis, an iron-driven cell death process worsened by ROS accumulation, through ferritin-based or ferrostatin-loaded nanoparticles may prevent lipid peroxidation and tissue damage [18]. This was specifically the focus of the Feng group who constructed nanoparticles filled with rutin. They subsequently found in both an in vitro and ex vivo study that these particles were able to congregate in renal tissue after IRI, but also protected against ferroptosis and cellular damage through removal of ROS [65]. Mitophagy-targeting nanoparticles also help to remove dysfunctional mitochondria, reducing oxidative stress and improving cell survival [18]. Advanced dual-functional systems, such as t-PA@iRNP, combine ROS scavenging with thrombolysis to prevent clot-induced IRI, particularly in stroke and myocardial infarction [64]. These nanomedicine strategies show strong potential in minimizing IRI-related damage across multiple organ systems, including the kidney, but have yet to progress to clinical trials.

### 3.7. Hydrogen Sulfide

Hydrogen sulfide (H₂S) is a potent therapeutic agent for mitigating IRI by targeting oxidative stress, inflammation, and mitochondrial dysfunction. As a gasotransmitter, H₂S protects against IRI through multiple mechanisms, including suppressing pro-inflammatory cytokines (IL-1β, TNF-α), modulating ER stress-induced autophagy, and preserving mitochondrial function [66]. H₂S donors such as sodium hydrosulfide (NaHS) and AP39 improve renal function by enhancing oxygenation, reducing apoptosis, and lowering creatinine and blood urea nitrogen levels [66]. Additionally, H₂S inhibits ferroptosis and reduces oxidative damage by downregulating NOX4, a key enzyme in ROS production [67]. In neuronal tissue, inhaled H₂S enhances cell survival by promoting anti-apoptotic signaling (Bcl-2) while inhibiting pro-apoptotic pathways (Bax, NF-κB) [67]. In renal IRI, cystathionine γ-lyase (CSE)-produced H₂S plays a crucial role in protecting against damage by regulating ER stress-induced autophagy [65]. A C57/BL6 mouse model showed that NaHS pretreatment significantly improved renal function by reducing serum creatinine, urea nitrogen levels, and ER stress markers (LC3II/I, Beclin-1), whereas the inhibition of CSE exacerbated renal damage [65].

Sodium thiosulfate (STS), a hydrogen sulfide (H₂S) donor, has demonstrated significant protective effects against IRI, particularly in kidney transplantation. Studies have shown that supplementing University of Wisconsin preservation solution with STS improves renal graft function by reducing apoptosis, inflammation, and acute tubular necrosis (ATN) [68]. In a rat model of kidney transplantation, STS supplementation decreased serum creatinine and blood urea nitrogen levels while enhancing urine output and osmolality, indicating improved graft viability [69]. Additionally, STS treatment at 10 °C enhanced the survival of proximal tubular epithelial cells by reducing apoptotic and necrotic cell death, further confirming its cytoprotective role [69]. These findings were most recently replicated in a porcine model which also showed less ATN on histopathological analysis in conjunction with greater urine output in porcine grafts treated with STS [70]. By modulating oxidative stress and preserving mitochondrial function, STS offers a clinically viable approach to mitigating cold IRI and improving transplant outcomes. These findings highlight H₂S and, by extension, STS as a promising therapeutic strategy for reducing IRI-related injury, as multiple models have shown an improvement in transplantation outcomes.

## 4. Conclusions

ESRD is a serious pathological process that affects various organ systems resulting in high mortality rates if left untreated. With an aging population and a parallel rise in comorbidities, the prevalence of ESRD continues to rise and a better understanding of how to enhance long-term graft survival for kidney transplants is essential. IRI, a phenomenon commonly seen in transplantation, has been shown to be an important factor in a graft’s function and survival.

IRI is a highly multifactorial and interconnected process primarily involving oxidative damage, inflammation, the immune response, and various programmed cell death mechanisms. The initial hypoxic injury and subsequent ROS formation from reperfusion lead to excess mtDNA damage and mitochondrial destruction, catalyzing the production of cytokines to drive inflammation and the subsequent immune response. In conjunction, the propagative nature of non-apoptotic programmed cell death mechanisms can also produce pro-inflammatory signals. These mechanisms can ultimately form a positive feedback system that causes the large-scale destruction of the graft.

Interrupting this process at any point is a promising target for protecting transplanted kidneys. Blocking ROS formation and ROS sequestration are especially attractive methods due to their role as one of the earliest drivers of IRI. Beyond this, multiple other modalities of treatment like miRNAs, peptides, and gasotransmitters are being explored due to their roles in inhibiting inflammation and cell death, which initiate many of the downstream consequences in IRI as they propagate. Though many of these remain in the pre-clinical stage, they remain a hotbed of innovative strategies in further attempts to preserve transplanted kidney graft function. With this review, we hope to have consolidated some of the research in regard to the mechanisms of IRI and its relevance to kidney transplantation as well as how it informs the current research on treatments to ameliorate it.

## Figures and Tables

**Table 1:** Graft survival and ischemia time stratified by donor type.

Type of Donors	Living	Circulatory Death	Brain Death
Graft survival (1 year average)	97.8%	79–94.3%	84–92%
Graft survival (5 year average)	86.5%	58.8–85.9%	73–84.5%
Incidence of delayed graft function	NA	49–73%	25–27%
Median ischemia time (in hours)	1–2	4–46	3–45
Median graft survival (in years)	19.2	9.7	NA
NA: (data) not available			

## Data Availability

No new data were created for this study. Studies cited in this review may be found in the references section.

## Abbreviations

The following abbreviations are used in this manuscript:

ESRD	End-stage renal disease
DCD	Donor after circulatory death
DBD	Donor after brain death
IRI	Ischemia–reperfusion injury
ROS	Reactive oxygen species
NOS	Nitric oxide synthase
Drp1	Dynamin-related protein 1
mtDNA	Mitochondrial DNA
mtROS	Mitochondrial reactive oxygen species
TFAM	Mitochondrial transcription factor A
ATP	Adenosine triphosphate
PAF	Platelet-activating factor
PAF-LPL	Platelet-activating factor phospholipids
AKI	Acute kidney injury
CKD	Chronic kidney disease
FasL	Fas ligand
SCS	Static cold storage
MP	Machine perfusion
DGF	Delayed graft function
eGFR	Estimated glomerular filtration rate
miRNA	MicroRNA
TSP-1	Thrombospondin-1
H_2_S	Hydrogen sulfide
STS	Sodium thiosulfate
ATN	Acute tubular necrosis
